# The Incidence and Outcomes of Major Limb Amputation in New Zealand from 2010 to 2021

**DOI:** 10.3390/jcm13133872

**Published:** 2024-06-30

**Authors:** Odette Hart, Oliver Bernau, Manar Khashram

**Affiliations:** 1 Department of Surgery, Faculty of Medical and Health Sciences, The University of Auckland, Auckland 1023, New Zealand; 2 Department of Vascular and Endovascular Surgery, Waikato District Health Board, Hamilton 3204, New Zealand

**Keywords:** epidemiology, peripheral artery disease, chronic limb-threatening ischaemia, mortality, survival, diabetes mellitus, lower extremity amputation

## Abstract

**Background**: Major limb amputation (MLA) can be a common outcome due to severe peripheral artery disease (PAD) and diabetic foot disease (DFD), and it carries a significant mortality burden. In New Zealand (NZ), there is little documentation of the incidence rate and mortality after MLA. The aim was to report the national crude and standardised rates and the mortality post MLA. **Methods**: This retrospective observational study included all MLAs that occurred within NZ from 1/1/2010 to 31/12/2021 due to DFD and/or PAD. Two national databases (National Minimum Dataset and the Australasian Vascular Audit) were utilised. The crude rates were calculated as cases per 100,000 in the NZ population per year including all ages (using the 2013 and 2018 NZ census figures). The age-standardised rates used the World Health Organization standard population. Post-operative mortality was calculated from the date of first hospitalisation for MLA. **Results**: From 2010 to 2021, there were 5293 MLA procedures in 4242 patients. On average, there were 8.5 MLAs per week and 441.1 MLAs annually. The overall crude rate was 9.44 per 100,000, and the standardised rate was 6.12 per 100,000. Over the 12 years, the crude rate decreased by 22% (*p* < 0.001), and the standardised rate decreased by 20.4% (*p* < 0.001). After MLA, the 30-day and 1-year mortality was 9.5% and 29.6%, respectively. From 2010 to 2021, the relative reduction in 30-day mortality was 45.1% (*p* < 0.001), and the reduction in 1-year mortality was 24.5% (*p* < 0.001). Increasing age, female sex and end-stage renal failure were predictors of 30-day and 1-year mortality. **Conclusions**: A considerable number of MLAs occur in NZ, with substantial perioperative mortality; however, the national incidence rates and mortality have improved over the last 12 years. This data might serve as benchmark to further reduce MLAs and improve patient outcomes.

## 1. Introduction

International data report that major limb amputation (MLA) is associated with high perioperative and 1-year mortality. Peripheral artery disease (PAD) and diabetic foot disease (DFD) are leading causes of MLA. PAD is an atherosclerotic arterial disease that results in impaired perfusion to the lower limb and spans a spectrum of presentations, ranging from asymptomatic to atypical leg symptoms, typical intermittent claudication and chronic limb-threatening ischaemia (CLTI) [1,2]. DFD is a frequent complication of diabetes mellitus (DM) and is defined as PAD, peripheral neuropathy, infection, ulceration, neuro-osteoarthropathy or gangrene of the foot in a person with DM [1].

The worldwide incidence of MLAs is 3.6 to 68.4 per 100,000, and the rate is significantly higher in the diabetes mellitus (DM) population (5.6 to 600 per 100,000) [3,4,5]. In New Zealand (NZ), the 2016 crude rate of MLAs was reported to be 8 per 100,000 individuals, decreasing from 12 per 100,000 in 2005 [6]. The VASUNET study (a collaboration of vascular registries from Europe and Australasia) suggested that from 2010 to 2014, the mean annual NZ incidence of MLAs was 7.2 per 100,000 [7]. This reported that NZ had the lowest incidence of MLAs among the countries reviewed, including Australia (8.3 per 100,000) and European countries (including the United Kingdom, at 8.8 per 100,000, and Germany, at 18.7 per 100,000). Furthermore, these studies reported that 54.5–58% of patients that underwent MLAs in NZ had DFD [6,7]. However, beyond this, in NZ, there is little documentation of the rate of MLA, and the national 30-day and 1-year mortality rates for MLA have not been described.

Previous studies reporting NZ’s rate of MLA have relied on event data from a single source [6,7]. Epidemiological studies utilising large administrative databases are subject to a lack of individual patient data, which may result in the misreporting of MLA rates or associated comorbidities. Hence, it is important to use methods that improve the data completeness by limiting missed events. One strategy is to combine several national databases to increase the accuracy and quality of event capture. Combining databases might provide greater knowledge of the contemporary epidemiology of MLA within NZ. The aim of this study was to report the incidence of MLA and the 30-day and 1-year mortality post MLA.

## 2. Materials and Methods

### 2.1. Study Design

This retrospective observational study used two national databases to create a “combined dataset” of all MLAs that occurred within NZ over the period from 1 January 2010 to 31 December 2021. The inclusion criterion was MLA indicated due to DFD and/or PAD. The exclusion criteria were (Appendix A) as follows:

- MLA indicated due to malignancy or trauma with “accident_code” (as per International Classification of Diseases (ICD-10-AM)
- Re-amputation of a stump or soft tissue and/or bone debridement of a stump (as per the ICD-10-AM procedure codes)
- All minor lower limb amputations (as per the ICD-10-AM procedure codes).

Ethics approval for this study was granted by the New Zealand Central Health and Disability Ethics Committee (20/CEN/122) and the Waikato District Health Board Research Department (RDO020044). Given the long retrospective nature, consent was waived. Reporting of this observational study followed the Strengthening the Reporting of Observational Studies in Epidemiology (STROBE) guidelines [8].

### 2.2. Study Protocol and Data Collection

Two of the national databases that collect information on MLA within NZ are the National Minimum Dataset (NMDS) and the Australasian Vascular Audit (AVA). The NMDS is a large administrative dataset comprising in-patient hospitalisations and day-patient health service provisions from private and public hospitals, dating from 1988 [9]. The AVA is an audit database implemented by the Australia and New Zealand Society for Vascular Surgery (ANZSVS) capturing all open surgical and endovascular procedures as entered by Australian and NZ vascular surgeons, dating from 2010 [10].

AVA data process: The AVA data were extracted for all NZ MLA procedures that occurred with a discharge date from 1 January 2010 to 31 December 2021 at all ages. MLAs with an indication of ischaemia, sepsis, infection, rest pain, or ulcers (arterial and non-arterial) were kept, and MLAs due to trauma and malignancy were excluded. There was a manual review to exclude MLA revisions; however, below-knee amputation (BKA) conversions into above-knee amputation (AKA) were included. A manual review occurred to capture bilateral MLAs during the same operation.

NMDS data process: Health New Zealand provided data from the NMDS. Data were requested for all hospitalisations with any procedure or diagnosis code relating to MLA or PAD according to the ICD-10-AM’s 6th and 8th Editions, with a discharge date from 1 January 2010 to 31 December 2021 and at all ages. Stump revisions and re-amputations of stumps using ICD-10-AM-procedure codes and malignancy and trauma (only if a concurrent “accident flag” code was present) using ICD-10-AM-diagnosis codes were excluded. A manual review occurred to capture bilateral MLAs during the same operation.

Combined dataset process: NMDS events were compared against AVA operations. The first matching process occurred by matching the patients’ unique National Health Indexes (NHIs), procedure dates and the sites of the MLAs. Procedures were considered equivalent if the difference between the procedure dates was ≤7 days apart to account for differences in the data accuracy. In the combined database, all the NMDS events were included, and any AVA procedures that were not found in the first match with the NMDS data were added. A second match via manual review occurred for the added AVA procedures to exclude any AVA procedures with overlapping or similar event dates (a manually confirmed procedure between the NMDS and AVA to ensure the same MLA type). For AVA procedures for which there was no NMDS event present, a manual review of the NHIs, DOBs and operation/admission dates occurred to ensure the NHIs were not incorrectly entered into AVA. Data from December 2021 and January 2022 were manually reviewed to capture and match cases to account for data inaccuracies during the final month of the study period. Patients were included more than once if they underwent multiple MLAs. Diagnoses within the AVA and NMDS were limited to patient encounters during the study interval, and there was no lookback period.

The combined dataset was utilised for national epidemiology of MLA. To establish the crude rates of MLA, national population counts were obtained from 2013 and 2018 census data of the usually resident population, retrieved from the national census database [11]. The AVA risk factors were grouped with NMDS diagnosis codes as follows: ischaemic heart disease (IHD:I20-I25), DM (E10, E11, E13, E14), hypertension (I10-I13, I15) and smoking (current: Z716, Z720, F17, ex: Z8643). Risk factors were considered present if they were in either the AVA or NMDS data.

### 2.3. Definitions

MLA procedures were defined as “any resection proximal to the ankle”, comprising below-knee amputation (BKA), through-knee amputation (TKA), above-knee amputation (AKA), amputation at the hip (or hip disarticulation) and hindquarter amputation [1]. The presence of DM was defined as haemoglobin A1c (HbA1c) greater than 48 mmol/mol. End-stage renal failure (ESRF) was defined as a decline in kidney function to a level at which either dialysis or kidney transplantation was required. Ischaemic heart disease (IHD) was defined as a history of cardiac symptoms secondary to coronary artery stenosis and hypertension, defined as a history of hypertension diagnosis (blood pressure ≥ 130/80 mmHg). Deprivation was defined using NZDep, which estimates the relative socioeconomic deprivation for areas on a scale from 1 (least deprived) to 10 (most deprived) [12]. The NZ deprivation (NZDep) index was prioritised as 2018 > 2013 > 2006 if the prior census year was blank. If it was not listed for any year, NZDep was considered not available (*n* = 21). The date of death was censored at 9 June 2022.

### 2.4. Statistical Analysis

Data were imported into Excel software version 2406 (Microsoft, WA, USA), and statistical analysis was performed using SPSS version 28 (IBM Corp., Armonk, NY, USA). Continuous data are summarised as medians (IQRs) or as means (SD) where appropriate, and categorical data are summarised as percentages. The Chi-squared test was used to compare categories, and a *p* value < 0.05 was considered significant. Each MLA procedure (e.g., first or second MLA) was analysed as an individual event. The MLA procedures were analysed as the number of procedures per week and the crude rates (cases per 100,000 of the NZ population per year) including patients of all ages. The 2013 and 2018 NZ census figures were used as the denominators to calculate the crude rates. The World Health Organisation (WHO) standard population was used for age standardisation of the MLA rates. Linear regression was used to assess the change in the MLA rates. The 30-day and 1-year post-operative mortality was calculated using the first hospitalisation for MLA and was based on the date of the operation. Variables in the univariate analysis with *p* < 0.2 were added into the multivariate analysis for mortality at 30 days and 1 year using logistic regression (odds ratio, OR).

## 3. Results

**Figure 1:**
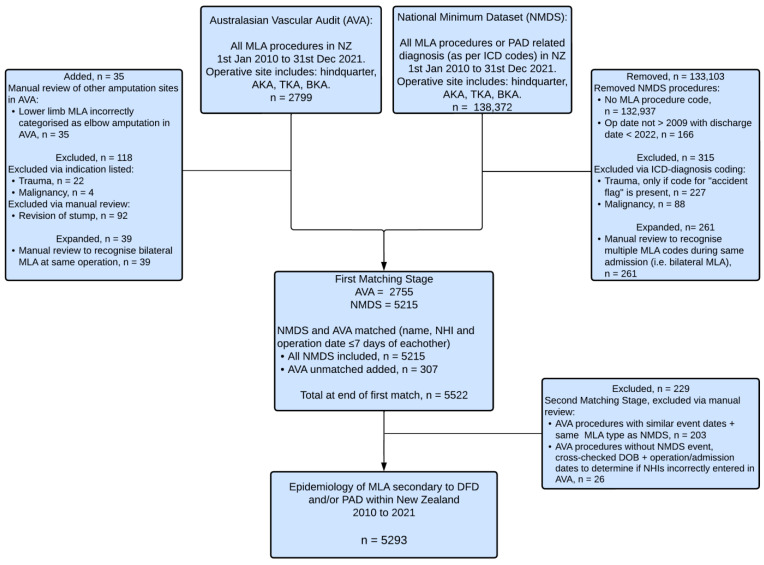
Flowchart of data inclusion in the study.

From 1 January 2010 to 31 December 2021, there were 5293 MLA procedures in 4242 patients with DFD and/or PAD, with 5215 (98.5%) identified in the NMDS and an additional 78 (1.5%) from the AVA (Figure 1). On average, 8.5 MLAs occurred per week and 441.1 MLAs occurred each year in NZ. The majority of patients (78.2%) underwent one MLA procedure; however, 21.8% of patients had more than one MLA owing to bilateral amputation or below- to above-knee conversion (Table 1). Patients with DM underwent a higher number of multiple MLAs compared to those without DM (DM: 25.7%; no DM: 10.2%; *p* < 0.001).

**Figure 2:**
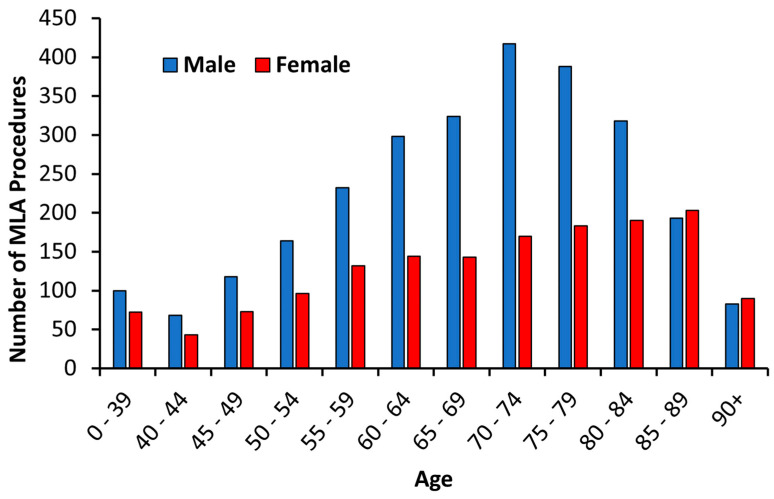
The age of men and women at their first MLA procedure during 2010–2021. MLA, major limb amputation.

The age stratification showed the number of MLAs increased as age increased for men and women (left-skewed, W = 0.96, *p* < 0.001; Figure 2). Patients that lived in high-deprivation areas were more likely to undergo MLAs than those from the least deprived areas; thus, from 2010 to 2021, 60.2% (2490/4185) of the patients that underwent MLA resided in areas with an NZDep of 7 to 10 (higher deprivation). Overall, smoking history (65.3%), hypertension (60.6%), DM (57.2%), IHD (37.1%) and ESRF (30.3%) were common comorbidities in patients that underwent MLAs.

**Figure 3:**
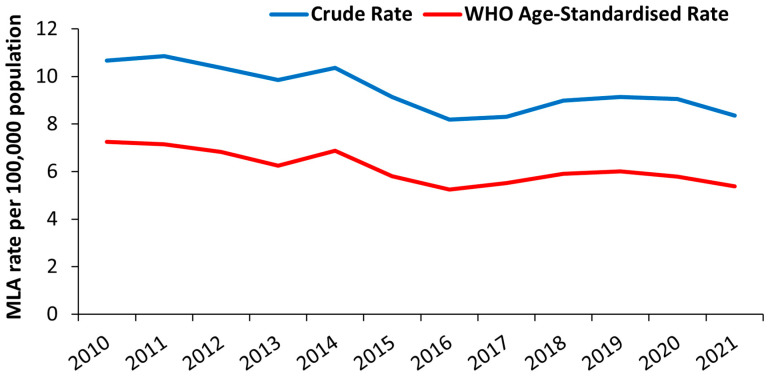
Line graph of annual MLA crude rate and annual WHO age-standardised rate within NZ from 2010 to 2021. MLA, major limb amputation; WHO, World Health Organization.

The 12-year average crude rate of MLAs was 9.44 per 100,000 individuals (Table 2). The yearly number of MLAs remained relatively stable (range 386–476); thus, due to the growth of the NZ population, the crude rate fell from 10.67 per 100,000 in 2010 to 8.35 per 100,000 in 2021 (decreased by 22%; *p* < 0.001, Figure 3). The WHO age-standardised rate also decreased by 20.4% (*p* < 0.001). Age stratification demonstrated the crude rate of MLAs increased with age (0–19 years: 0.23 per 100,000; 20–39 years: 1.07 per 100,000; 40–59 years: 7.79 per 100,000; 60–79 years: 28.34 per 100,000; ≥80 years 64.89 per 100,000).

**Figure 4:**
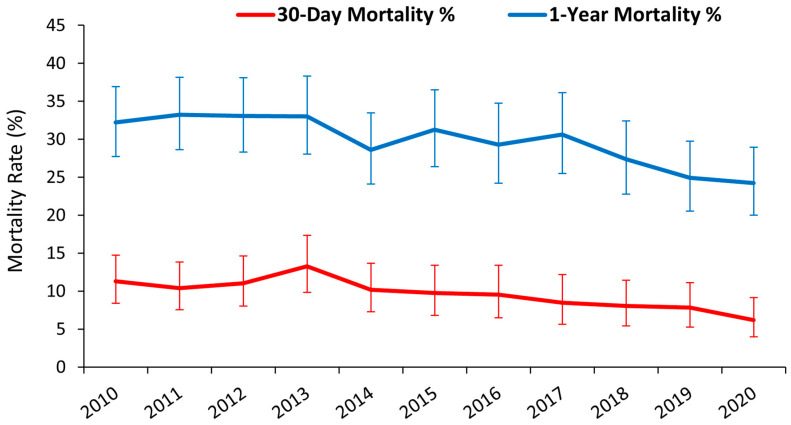
Line graph showing the 30-day and 1-year all-cause mortality rates per hospitalisation for MLA from 2010 to 2020. Error bars are 95% confidence intervals.

National mortality data were complete for at least one-year post MLA. After the first MLA hospitalisation, the mean 30-day all-cause mortality was 9.5%, and the mean 1-year all-cause mortality was 29.6% (Table 3). From 2010 to 2020, the 30-day mortality rate improved from 11.3% (95% CI 8.4–14.7) to 6.2% (95% CI 4–9.2), with a relative risk reduction for 30-day mortality of 45.1% (*p* < 0.001, Figure 4). The 1-year mortality rate improved from 32.2% (95% CI 27.7–36.9) to 24.3% (95% CI 20–29), with a relative risk reduction for 1-year mortality of 24.5% (*p* < 0.001).

There were 477 patients that died ≤ 30 days post MLA. The univariate analysis demonstrated that of the variables examined, age, sex and the presence of ESRF were associated with mortality ≤ 30 days post MLA (*p* < 0.001). The multivariate logistic regression showed that the probability of death ≤30 days post MLA increased with older age (OR 1.02, 95% CI 1.01–1.03, *p* < 0.001), female sex (OR 1.46, 95% CI 1.20–1.77, *p* < 0.001) and ESRF (OR 2.16, 95% CI 1.78–2.64, *p* < 0.001; Table 4).

After excluding patients with mortality ≤ 30 days, there were 3765 patients in the univariate analysis for mortality ≤ 1 year post MLA. This demonstrated that 907 patients died ≤ 1 year post MLA, and of the variables examined, age, DM and ESRF were significantly related to mortality within ≤1 year post MLA (*p* < 0.001). Multivariate logistic regression showed that the probability of death ≤1 year post MLA was increased with older age (OR 1.04, 95% CI 1.03–1.05, *p* < 0.001) and ESRF (OR 2.14, 95% CI 1.80–2.55, *p* < 0.001) but not DM (OR 1.19 (0.99–1.44, *p* = 0.06; Table 5).

## 4. Discussion

From 2010 to 2021, the average number of limbs lost due to DFD and/or PAD was 8.5 limbs per week and 444.1 limbs per year. NZ’s average crude rate of MLAs for all ages was 9.44 per 100,000. The 2016 MLA crude rate (8.19 per 100,000) was similar to the MLA crude rate reported in Gurney et al. 2016, at 8 per 100,000 [6]. However, the crude rates produced by VASCUNET under-reported NZ’s MLA rates by 28.8% (2010), 30% (2011), 29.6% (2012), 24.9% (2013) and 41.1% (2014) compared to our study [7]. This occurred despite VASCUNET using the same methodology, with each MLA procedure as an individual event, and is likely due to the utilisation of the AVA only as a single data source.

The crude rate of MLAs in NZ decreased by 22% from 2010 to 2021. This aligns with another NZ study (3% decrease per year from 2005 to 2016 [6]) and international studies from England, reporting a 20% decrease from 2003 to 2013 [13], and Germany, reporting a 30.9% decrease from 2005 to 2014 [14], and the VASCUNET figures for other countries [7]. However, some countries have not experienced an improvement in MLA rates [15]. There is substantial variation in the MLA rates recorded internationally, and caution is required when comparing rates due to differing methodologies, incomplete datasets, the administration of healthcare (public versus private), a lack of standardised diagnostic codes, procedures being carried out by non-vascular specialities and the fact that every patient has the potential for two limbs to be amputated at differing levels [5,7,16]. These factors, when combined with differences in the risk factors, within populations may cause wide variations in the MLA rates reported within or between countries.

This study demonstrated the MLA rate was highly related to NZ deprivation and male sex. This is in line with a previous study focused on MLA in DFD [17]. Young patients (<40 years) were also overrepresented in the MLA rates (4.1%). International studies reported a high morbidity burden (particularly with DM and ESRF) in young patients that underwent non-traumatic MLAs [18,19]. CVD comorbidities and risk factors were also frequent in the population undergoing MLAs; these included a history of smoking, hypertension, DM, IHD and ESRF. Understanding the underlying causes of the disparities within NZ that lead to an increased risk of amputation is pivotal for future gains to reduce the MLA rates [17,20]. There is a need for increased focus on prevention strategies, including early detection of DFD and PAD, with streamlined referral to podiatry and vascular surgery services, which will allow for early intervention for foot care and revascularisation, as well as the implementation of risk reduction measures and programs [21,22].

After MLA, the 30-day mortality was 9.5%, and the one-year mortality was 29.6%. These figures were higher than those in a recent study (2007–2018) from California, which comprised 26,669 patients that underwent MLAs and reported a 30-day mortality rate of 4.8% and a 1-year mortality rate of 12.47% post MLA [23]. However, it is similar to a cohort of DFD only that underwent MLAs in NZ, with a 30-day mortality of 11% [24]. The high portion of patients with DM in the national MLA rates (57.2%) may cause the national mortality due to MLAs to be more closely aligned with DM rates of mortality. During the 12 years reviewed, there was a significant improvement in the 30-day and 1-year mortality rates after first hospitalisation for patients that underwent MLAs. International data have also suggested that the 30-day and 1-year mortality rates have decreased in recent decades; hence, the contemporary literature demonstrates that patients may live longer after MLA than previously reported [23,25].

This study demonstrated that an older age and ESRF increased the probability of 30-day and one-year mortality post MLA. An increased odds of death for people with a history of renal disease after MLA has been well reported internationally [26]. Importantly, our study is novel in demonstrating that female sex increases the probability of mortality ≤ 30 days post MLA for patients in NZ. The recent literature from NZ reports that there is worse survival for women after surgical or percutaneous intervention for critical limb-threatening ischaemia (CLTI) within the Midland region of NZ [27]. Further research to elucidate the causes of a higher mortality risk for women with CLTI and in MLA within NZ is required.

One strength of this study is the assessment of national MLA rates using a combined database from two data sources to improve the data capture. Common weaknesses in the methodology for MLA epidemiology studies have been mitigated by defining the age group studied, the sources of data and the population age group of the denominator, defining MLA as amputation above the ankle, listing ICD codes and producing results that are standardised, including the production of age-specific results [16]. MLA is a preventable outcome of PAD and DFD; hence, the national MLA rates are of high importance to governing bodies. This study shows that prevention strategies could target reducing the MLA rates for young people, men and those with DM, while improvements in post-operative mortality may arise from focusing on women and those with ESRF. However, this study has limitations. Firstly, there may have been MLA procedures that were not entered into either the NMDS or the AVA and hence not represented in this study. Secondly, there was a risk of repeat procedures being captured in the data. This was reduced by using the unique patient identifier (NHIs) which are assigned to all healthcare data in NZ. There were also multiple manual reviews of the data to reduce data errors. Additionally, mortality was represented as the rate per first hospitalisation for MLA. It cannot be confirmed that this was the index MLA for each patient, as there was no lookback period; hence, this may have created a bias, with the inclusion of the second to fourth MLA nearer the 2010 time period potentially causing a higher mortality rate (if there is a difference in mortality between the first MLA and subsequent MLAs). Finally, this study captured major variables associated with MLA; however, this list of variables is not exhaustive, and it is possible there are factors associated with mortality after MLA that were not reviewed in this study.

## 5. Conclusions

MLA continues to be a frequent operation in NZ, and nationally, there are on average 8.5 MLAs every week. Higher deprivation in NZ was highly related to MLA, and young people (<40 years) were over-represented; hence, dedicated studies are required to correct the sources of inequality that lead to these outcomes. The national crude and standardised rates of MLA decreased over the 12 years reviewed. Hence, there is a need for investigation into the factors associated with decreased MLA rates so that further improvements in limb salvage can occur for DFD and PAD. Similar to other countries, the 30-day and 1-year post MLA mortality rates improved during the study period, although there was still considerable post-operative mortality associated. This study demonstrated that improvements in post MLA mortality rates may come from research and interventions on the outcome discrepancies for women and patients with ESRF. These data might serve as a benchmark for future research regarding national MLA rates and the mortality after MLA.

## Figures and Tables

**Table 1:** The demographics of patients that underwent MLAs from 2010 to 2021 within NZ.

Patient Characteristics			Patients, *n* = 4242 (%)	Missing (%)
Number of MLAs per person				
	1		3318 (78.2)	0 (0)
	2		805 (19)	
	3		111 (2.6)	
	4		8 (0.2)	
Age, median (IQR) years			70 (59–80)	
Age group				
	0–39		172 (4.1)	0 (0)
	40–49		302 (7.1)	
	50–59		624 (14.7)	
	60–69		909 (21.4)	
	70–79		1158 (27.3)	
	80–89		904 (21.3)	
	90+		173 (4.1)	
Sex				
	Male		2703 (63.7)	0 (0)
	Female		1539 (36.3)	
NZ Deprivation Index			4185	
	1–2		356 (8.5)	57 (1.3)
	3–4		584 (14.0)	
	5–6		766 (18.3)	
	7–8		1099 (26.3)	
	9–10		1380 (339)	
Comorbidities				
	Diabetes Mellitus		2962 (69.8)	0 (0)
	ESRF		1278 (30.3)	20 (0.5)
	Hypertension		2845 (67.1)	0 (0)
	Ischaemic Heart Disease		1751 (41.3)	0 (0)
	Smoking			
		Non-Smoker	1212 (28.6)	0 (0)
		Ex-Smoker	2122 (50.0)	
		Current	908 (21.4)	

IQR, interquartile range; NZ, New Zealand; ESRF, end-stage renal failure.

**Table 2:** The crude and WHO age-standardised rates of MLAs within NZ from 2010 to 2021.

Year	Total MLA	NZ Population	Crude Rate, per 100,000	Age-Standardised (WHO) Rate, per 100,000
2010	464	4,350,670	10.67	7.26
2011	476	4,383,990	10.86	7.15
2012	457	4,408,040	10.37	6.83
2013	438	4,442,100	9.86	6.24
2014	468	4,516,470	10.36	6.87
2015	421	4,609,370	9.13	5.8
2016	386	4,714,120	8.19	5.24
2017	400	4,813,580	8.31	5.52
2018	440	4,900,620	8.98	5.92
2019	455	4,979,170	9.14	6.02
2020	461	5,090,170	9.06	5.78
2021	427	5,111,310	8.35	5.38
**Total**	**5293**	**5,111,300**	**9.44**	**6.12**

MLA; major limb amputation; NZ, New Zealand; WHO, World Health Organization.

**Table 3:** The all-cause 30-day and 1-year mortality rates per first hospitalisation for MLA from 2010 to 2020.

Year	Patients	Deaths (30 Days)	30-Day Mortality Rate% (95% CI)	Deaths (1 Year)	1-Year Mortality Rate% (95% CI)
2010	416	47	11.3 (84–14.7)	134	32.2 (27.7–36.9)
2011	394	41	10.4 (7.6–13.9)	131	33.3 (28.6–38.1)
2012	372	41	11 (8–14.7)	123	33 (28.3–38.1)
2013	339	45	13.3 (9.9–17.4)	112	33 (28.1–38.3)
2014	374	38	10.2 (7.3–13.7)	107	28.6 (24.1–33.5)
2015	339	33	9.7 (6.8–13.4)	106	31.3 (26.4–36.5)
2016	304	29	9.5 (6.5–13.4)	89	29.3 (24.2–36.1)
2017	307	26	8.5 (5.6–12.2)	94	30.6 (25.5–36.1)
2018	347	28	8.1 (2.4–11.5)	95	27.4 (22.8–32.4)
2019	357	28	7.8 (5.3–11.1)	89	24.9 (20.5–29.8)
2020	371	23	6.2 (4–9.2)	90	24.3 (20–29)
**Total**	**3920**	**379**	**9.7 (8.8–10.6)**	**1170**	**29.8 (28.4–31.3)**

CI, confidence interval.

**Table 4:** Logistic regression for mortality ≤ 30 days for patients that underwent major limb amputation (MLA).

				Univariate		Multivariate	
Variable		Total	Death ≤ 30 Days	OR (95% CI)	*p* Value	OR (95% CI)	*p* Value
Age Group		4242	477	1.02 (1.01–1.03)	<0.001	1.02 (1.01–1.03)	<0.001
Sex	Male	2703	264 (9.8)	Ref		Ref	
	Female	1539	213 (13.8)	1.48 (1.22–1.80)	<0.001	1.46 (1.20–1.77)	<0.001
DM	No	1280	124 (9.7)	Ref		Ref	
	Yes	2962	353 (11.9)	1.26 (1.02–1.57)	0.04	1.00 (0.78–1.27)	0.98
HTN	No	1397	149 (10.7)	Ref			
	Yes	2845	328 (11.5)	1.09 (0.89–1.34)	0.40		
IHD	No	2491	260 (10.4)	Ref		Ref	
	Yes	1751	217 (12.4)	1.21 (1.00–1.47)	0.05	1.20 (0.98–1.46)	0.08
ESRF	No	2944	263 (8.9)	Ref		Ref	
	Yes	1278	211 (16.5)	2.02 (1.66–2.45)	<0.001	2.16 (1.78–2.64)	<0.001
Smoking	Non-Smoker	1212	149 (12.3)	Ref	0.20		
	Ex	2122	239 (11.3)	0.91 (0.73–1.13)	0.37		
	Current	908	89 (9.8)	0.76 (0.59–1.02)	0.07		
NZ Deprivation	1–2	356	41 (11.5)	Ref	0.88		
	3–4	584	66 (11.3)	0.98 (0.65–1.48)	0.92		
	5–6	766	80 (10.4)	0.90 (0.60–1.34)	0.59		
	7–8	1109	121 (10.9)	0.94 (0.65–1.37)	0.75		
	9–10	1380	164 (11.9)	1.04 (0.72–1.49)	0.85		

OR, odds ratio; DM, diabetes mellitus; HTN, hypertension; IHD, ischaemic heart disease; ESRF, end-stage renal failure; NZ, New Zealand.

**Table 5:** Logistic regression for mortality ≤ 1 year (after removal of mortality ≤ 30 days) for patients that underwent a major limb amputation (MLA).

				Univariate		Multivariate	
Variable		Total	Death ≤ 1 Year	OR (95% CI)	*p* Value	OR (95% CI)	*p* Value
Age Group		3765		1.04 (1.03–1.04)	<0.001	1.04 (1.03–1.05)	<0.001
Sex	Male	2439	580 (23.8)	Ref			
	Female	1326	327 (24.7)	1.05 (0.90–1.23)	0.55		
DM	No	1156	235 (20.3)	Ref		Ref	
	Yes	2609	672 (25.8)	1.36 (1.45–1.61)	<0.001	1.19 (0.99–1.44)	0.06
HTN	No	1248	296 (23.7)	Ref			
	Yes	2517	611 (24.3)	1.03 (0.88–1.21)	0.71		
IHD	No	2231	532 (23.8)	Ref			
	Yes	1534	375 (24.4)	1.03 (0.89–1.20)	0.67		
ESRF	No	2681	358 (33.6)	Ref			
	Yes	1067	547 (20.4)	1.97 (1.68–2.31)	<0.001	2.14 (1.80–2.55)	<0.001
Smoking	Non-Smoker	1063	264 (24.8)	Ref	0.03		
	Ex	1883	475 (25.2)	1.02 (0.86–1.22)	0.81		
	Current	816	168 (20.5)	0.78 (0.63–0.97)	0.03		
NZ Deprivation	1–2	315	66 (21)	Ref	0.47		
	3–4	518	126 (24.3)	1.21 (0.87–1.70)	0.26		
	5–6	686	166 (24.2)	1.20 (0.87–1.66)	0.26		
	7–8	988	256 (25.9)	1.02 (0.97–1.79)	0.08		
	9–10	1216	288 (23.7)	2.17 (0.87–1.58)	0.31		

OR, odds ratio; DM, diabetes, mellitus; HTN, hypertension; IHD, ischaemic heart disease; ESRF, end-stage renal failure; NZ, New Zealand.

## Data Availability

Due to patient privacy, the datasets generated and analyzed during the study are not publicly available.

## Appendix A

ICD-10 codes for included diagnoses and excluded diagnoses.

**Table A1:** Diagnosis codes included (PAD codes).

I7020	Unspecified atherosclerosis of native arteries of extremities
I7021	Atherosclerosis of native arteries of extremities with intermittent claudication
I7022	Atherosclerosis of native arteries of extremities with rest pain
I7023	Atherosclerosis of native arteries of right leg with ulceration
I7024	Atherosclerosis of native arteries of left leg with ulceration
I7025	Atherosclerosis of native arteries of other extremities with ulceration
I7026	Atherosclerosis of native arteries of extremities with gangrene
I7029	Other atherosclerosis of native arteries of extremities
E1051	Type 1 diabetes mellitus with peripheral angiopathy, without gangrene
E1052	Type 1 diabetes mellitus with peripheral angiopathy, with gangrene
E1151	Type 2 diabetes mellitus with peripheral angiopathy, without gangrene
E1152	Type 2 diabetes mellitus with peripheral angiopathy, with gangrene
E1351	Other specified diabetes mellitus with peripheral angiopathy, without gangrene
E1352	Other specified diabetes mellitus with peripheral angiopathy, with gangrene
E1451	Unspecified diabetes mellitus with peripheral angiopathy, without gangrene
E1452	Unspecified diabetes mellitus with peripheral angiopathy, with gangrene

**Table A2:** Procedure codes included (MLA codes).

4436700	Amputation Above Knee
4436701	Disarticulation at Knee
4436702	Amputation Below Knee
4437000	Amputation at Hip
4437300	Hindquarter Amputation

**Table A3:** Related procedure codes excluded.

4437600	Re-Amputation of Amputation Stump
3002300	Debridement of Amputation Stump Involving Soft Tissue
3002301	Debridement of Amputation Stump Involving Soft Tissue and Bone
4433800	Amputation of Toe
4435800	Amputation of Toe, Including Metatarsal Bone
4436100	Disarticulation Through Ankle
4436400	Midtarsal Amputation
4436400	Transmetatarsal Amputation
9055700	Disarticulation Through Toe

## References

[1] van Netten J.J., Bus S.A., Apelqvist J., Chen P., Chuter V., Fitridge R., Game F., Hinchliffe R.J., Lazzarini P.A., Mills J. (2023). Definitions and criteria for diabetes-related foot disease (IWGDF 2023 update). Diabetes Metab. Res. Rev..

[2] Patel M.R., Conte M.S., Cutlip D.E., Dib N., Geraghty P., Gray W., Hiatt W.R., Ho M., Ikeda K., Ikeno F. (2015). Evaluation and treatment of patients with lower extremity peripheral artery disease: Consensus definitions from Peripheral Academic Research Consortium (PARC). J. Am. Coll. Cardiol..

[3] Moxey P.W., Gogalniceanu P., Hinchliffe R.J., Loftus I.M., Jones K.J., Thompson M.M., Holt P.J. (2011). Lower extremity amputations—A review of global variability in incidence. Diabet. Med..

[4] Fosse S., Hartemann-Heurtier A., Jacqueminet S., Ha Van G., Grimaldi A., Fagot-Campagna A. (2009). Incidence and characteristics of lower limb amputations in people with diabetes. Diabet. Med..

[5] Narres M., Kvitkina T., Claessen H., Droste S., Schuster B., Morbach S., Rumenapf G., Van Acker K., Icks A. (2017). Incidence of lower extremity amputations in the diabetic compared with the non-diabetic population: A systematic review. PLoS ONE.

[6] Gurney J.K., Stanley J., York S., Sarfati D. (2018). Lower-limb amputation in New Zealand: Temporal changes and the role of diabetes mellitus. N. Z. Med. J..

[7] Behrendt C.A., Sigvant B., Szeberin Z., Beiles B., Eldrup N., Thomson I.A., Venermo M., Altreuther M., Menyhei G., Nordanstig J. (2018). International variations in amputation practice: A VASCUNET report. Eur. J. Vasc. Endovasc. Surg..

[8] von Elm E., Altman D.G., Egger M., Pocock S.J., Gotzsche P.C., Vandenbroucke J.P., Initiative S. (2007). Strengthening the Reporting of Observational Studies in Epidemiology (STROBE) statement: Guidelines for reporting observational studies. BMJ.

[9] Te Whatu Ora National Minimum Dataset (Hospital Events): New Zealand Government, 2023. https://www.tewhatuora.govt.nz/our-health-system/data-and-statistics/nz-health-statistics/national-collections-and-surveys/collections/national-minimum-dataset-hospital-events.

[10] Bourke B.M., Beiles C.B., Thomson I.A., Grigg M.J., Fitridge R. (2012). Development of the Australasian vascular surgical audit. J. Vasc. Surg..

[11] Stats N.Z. Population Statistics. Wellington: Statistics New Zealand Tatauranga Aotearoa, 2019. www.stats.govt.nz.

[12] Salmond C.E., Crampton P. (2012). Development of New Zealand’s deprivation index (NZDep) and its uptake as a national policy tool. Can. J. Public Health.

[13] Ahmad N., Thomas G.N., Gill P., Torella F. (2016). The prevalence of major lower limb amputation in the diabetic and non-diabetic population of England 2003-2013. Diab. Vasc. Dis. Res..

[14] Kroger K., Berg C., Santosa F., Malyar N., Reinecke H. (2017). Lower Limb Amputation in Germany. Dtsch. Arztebl. Int..

[15] Coman H.F., Stancu B., Andercou O.A., Ciocan R., Gherman C.D., Rusu A., Gavan N.A., Bondor C.I., Gavan A.D., Bala C.G. (2024). Five-year trends of vascular disease-related amputations in Romania: A retrospective database study. J. Clin. Med..

[16] Davies M., Burdett L., Bowling F., Ahmad N., McClennon J. (2019). The epidemiology of major lower-limb amputation in England: A systematic review highlighting methodological differences of reported trials. Diabet. Foot J..

[17] Gurney J.K., Stanley J., York S., Rosenbaum D., Sarfati D. (2018). Risk of lower limb amputation in a national prevalent cohort of patients with diabetes. Diabetologia.

[18] Chin J.W., Teague L., McLaren A.M., Mahoney J.L. (2013). Non traumatic lower extremity amputations in younger patients: An 11-year retrospective study. Int. Wound J..

[19] Geiss L.S., Li Y., Hora I., Albright A., Rolka D., Gregg E.W. (2019). Resurgence of diabetes-related nontraumatic lower-extremity amputation in the young and middle-aged adult U.S. population. Diabetes Care.

[20] Shin J.Y., Roh S.G., Sharaf B., Lee N.H. (2017). Risk of major limb amputation in diabetic foot ulcer and accompanying disease: A meta-analysis. J. Plast. Reconstr. Aesthet. Surg..

[21] Li Q., Birmpili P., Johal A.S., Waton S., Pherwani A.D., Boyle J.R., Cromwell D.A. (2022). Delays to revascularization for patients with chronic limb-threatening ischaemia. Br. J. Surg..

[22] Onofrei V.A., Ceasovschih A., Marcu D.T.M., Adam C.A., Mitu O., Mitu F. (2022). Mortality risk assessment in peripheral arterial disease-the burden of cardiovascular risk factors over the years: A single center’s experience. Diagnostics.

[23] Beeson S.A., Neubauer D., Calvo R., Sise M., Martin M., Kauvar D.S., Reid C.M. (2023). Analysis of 5-year Mortality following Lower Extremity Amputation due to Vascular Disease. Plast. Reconstr. Surg. Glob. Open.

[24] Gurney J.K., Stanley J., Rumball-Smith J., York S., Sarfati D. (2018). Postoperative death after lower-limb amputation in a national prevalent cohort of patients with diabetes. Diabetes Care.

[25] Moxey P.W., Hofman D., Hinchliffe R.J., Jones K., Thompson M.M., Holt P.J. (2010). Epidemiological study of lower limb amputation in England between 2003 and 2008. Br. J. Surg..

[26] Fortington L.V., Geertzen J.H., van Netten J.J., Postema K., Rommers G.M., Dijkstra P.U. (2013). Short and long term mortality rates after a lower limb amputation. Eur. J. Vasc. Endovasc. Surg..

[27] Hart O., Xue N., Davis-Havill B., Pottier M., Prakash M., Reimann S.A., King J., Xu W., Khashram M. (2022). The incidence of chronic limb-threatening ischemia in the Midland region of New Zealand over a 12-year period. J. Clin. Med..

